# Combination of Goniothalamin and Sol-Gel-Derived Bioactive Glass 45S5 Enhances Growth Inhibitory Activity *via* Apoptosis Induction and Cell Cycle Arrest in Breast Cancer Cells MCF-7

**DOI:** 10.1155/2022/5653136

**Published:** 2022-07-14

**Authors:** Siti Aishah Abu Bakar, Abdul Manaf Ali, Siti Noor Fazliah Mohd Noor, Shahrul Bariyah Sahul Hamid, Nur Asna Azhar, Noor Muzamil Mohamad, Nor Hazwani Ahmad

**Affiliations:** ^1^Department of Biomedical Science, Advanced Medical and Dental Institute, Universiti Sains Malaysia, Bertam 13200 Kepala Batas, Pulau Pinang, Malaysia; ^2^Faculty of Bioresources and Food Industry, Universiti Sultan Zainal Abidin, Besut Campus, 22200 Besut, Terengganu Darul Iman, Malaysia; ^3^Department of Dental Science, Advanced Medical and Dental Institute, Universiti Sains Malaysia, Bertam 13200 Kepala Batas, Pulau Pinang, Malaysia; ^4^Centralised Laboratory Management Center, Universiti Sultan Zainal Abidin, Besut Campus, 22200 Besut, Terengganu Darul Iman, Malaysia

## Abstract

**Background:**

Combination of natural products with chemically synthesised biomaterials as cancer therapy has attracted great interest lately. Hence, this study is aimed at investigating the combined effects of goniothalamin and bioactive glass 45S5 (GTN-BG) and evaluating their anticancer properties on human breast cancer cells MCF-7.

**Methods:**

The BG 45S5 was prepared using the sol-gel process followed by characterisation using PSA, BET, SEM/EDS, XRD, and FTIR. The effects of GTN-BG on the proliferation of MCF-7 were assessed by MTT, PrestoBlue, and scratch wound assays. The cell cycle analysis, Annexin-FITC assay, and activation of caspase-3/7, caspase-8, and caspase-9 assays were determined to further explore its mechanism of action.

**Results:**

The synthesised BG 45S5 was classified as a fine powder, having a rough surface, and contains mesopores of 12.6 nm. EDS analysis revealed that silica and calcium elements are the primary components of BG powders. Both crystalline and amorphous structures were detected with 73% and 27% similarity to Na_2_Ca_2_(Si_2_O_7_) and hydroxyapatite, respectively. The combination of GTN-BG was more potent than GTN in inhibiting the proliferation of MCF-7 cells. G0/G1 and G2/M phases of the cell cycle were arrested by GTN and GTN-BG. The percentage of viable cells in GTN-BG treatment was significantly lower than that in GTN. In terms of activation of initiator caspases for both extrinsic and intrinsic apoptosis pathways, caspase-8 and caspase-9 were found more effective in response to GTN-BG than GTN.

**Conclusion:**

The anticancer effect of GTN in MCF-7 cells was improved when combined with BG. The findings provide significant insight into the mechanism of GTN-BG against MCF-7 cells, which can potentially be used as a novel anticancer therapeutic approach.

## 1. Background

Breast cancer is the most frequently occurring cancer in women and the second most common cancer overall, which increases among women with over 2 million worldwide [1]. The treatment for breast cancer includes surgery and chemotherapy using drugs to kill fast-growing cancer cells throughout the body; however, the treatment can also destroy healthy cells. Researchers around the world are therefore continuously looking for better strategies to improve breast cancer treatment. Natural compound could be considered as a promising alternative to the present chemotherapeutic drug if it has a strong range of anticancer activity with less toxicity towards normal cells. Besides, the monotherapy approach to cancer treatment could be enhanced by combining the anticancer agents with other substances that have an additive or synergistic effect in the killing of cancer cells.

Bioactive glasses (BG) were first discovered by Larry Hench in 1969 with an original BG composition of 45% SiO_2_, 24.5% Na_2_O, 24.5% CaO, and 6% P_2_O_5_ (in weight percentages, wt.%), formed using the traditional melt method at high temperature (1300–1500°C) [2, 3]. Alternatively, the sol-gel method has the advantages of low processing temperature and ease of control of textural properties [4]. Besides, sol-gel-derived glasses contain fewer components than the melt-derived glasses and may exhibit increased bioactivity and absorbability due to their increased surface area provided by the inherent soluble nanoporous texture. A larger surface area significantly improves solubility of the material [5]. Despite BG's major application in bone tissue engineering, there is a growing interest in the potential use of sol-gel-derived BG in other ground-breaking biomedical applications including soft tissue repair, nerve regeneration, drug delivery, and cancer treatment [6–8]. Apart from being nontoxic and highly biocompatible, BGs have shown great potential for anticancer application because of their unique properties, including ease of doping with magnetic ions; elements such as samarium and iron, as well as anticancer drugs; and chemotherapy drugs, namely, imatinib and doxorubicin, which are released locally in a controlled manner [8–12].

Goniothalamin (GTN), a natural styryl lactone extracted from *Goniothalamus* sp., has been reviewed to possess anticancer effects on several types of cancer cells, including leukaemia and breast, lung, oral, cervical, colon, ovarian, pancreatic, and prostate cancer cells with less toxicity towards normal cells [13, 14]. Most GTN-treated cancer cells were reported to undergo various forms of cell death, most notably apoptosis [15–21]. However, limited studies have been conducted focusing on the combination of GTN with any substance to enhance GTN's inhibitory effects on cancer cells. To date, scarce information is available regarding the combination of GTN with BG. Therefore, this study was designed to explore the anticancer effects of combined GTN and BG (GTN-BG) towards MCF-7 cells.

## 2. Methods

### 2.1. Goniothalamin (GTN) Compound

The powdered form of GTN compounds isolated from the roots of *Goniothalamus macrophyllus* was kindly provided by Prof. Dr. Abdul Manaf Ali from Universiti Sultan Zainal Abidin (UniSZA). It was dissolved in dimethyl sulphoxide (DMSO) (Vivantis, USA) to obtain a stock solution of 10 mg/mL and deposited in aliquots at -20°C for future usage. The 60 *μ*g/mL working solution was prepared by diluting the stock solution with phosphate-buffered saline (PBS).

### 2.2. Synthesis and Characterisation of Bioactive Glass 45S5

The BG powders were synthesised using the sol-gel method by Aliaa et al. with slight modification [22]. Briefly, a total of 33.5 mL tetraethyl orthosilicate (TEOS) was added to 50 mL of 1 M of nitric acid and stirred at room temperature for 1 hour. A volume of 2.9 mL triethyl phosphate (TEP), (C_2_H_5_)_3_PO_4_, and 20.63 g of calcium nitrate tetrahydrate, Ca (NO_3_)2.4H_2_O, were added to the solution at a 45-minute interval. Then, 13.42 g of sodium nitrate, NaNO_3_, was added and stirred overnight. The solution was incubated in an oven at 31°C for 3 days to accelerate gelation. The resulting gel was aged by incubation in the oven for 2 days at 60°C and the subsequent drying process at the temperature of 110°C for another 2 days. The dried gel was placed in an alumina cup and was calcined at 700°C for 3 hours to obtain finer BG powder. The synthesised BG powders were ground and sieved to obtain particle size less than 38 *μ*m. The particle size of the BG powder was confirmed using a particle size analyzer (Mastersizer 3000, UK), with distilled water used as the dispersion medium at 2500 rpm. The physicochemical properties of synthesised BG 45S5 powders were characterised using BET, SEM/EDS, FTIR, and XRD. The surface area and pore size of the powder of BG were measured by a nitrogen adsorption technique known as the Brunauer, Emmel, and Teller (BET) method. Nitrogen adsorption-desorption isotherms at -196°C were obtained using a Micromeritics ASAP 2020 Analyzer, USA. The surface morphology of BG powder was observed using a scanning electron microscope (SEM) (Joel JSM 6360 LA, Japan). The phase purity and crystallinity of the synthesised powder were obtained from X-ray diffraction analysis (XRD) (Rigaku Miniflex-II, Japan), and the chemical groups in the BG structures were identified using Fourier transform infrared spectroscopy (FTIR) (IRTracer-100-Shimadzu, Japan). The BG-conditioned medium was prepared at stock concentration of 10 mg/mL in culture medium without serum and incubated in an incubator shaker for 4 hours at 37°C, followed by filtration using a 0.22 *μ*m syringe filter. Prior to the cell treatment, the BG-conditioned medium was incubated overnight at 37°C in a CO_2_ incubator (Shellab, USA).

### 2.3. Cell Culture

Breast adenocarcinoma cells (MCF-7, ATCC) and human bone marrow-derived mesenchymal stem cells (HMSC, Lonza) were kindly provided by the team members from AMDI, USM. The MCF-7 and HMSC cells were cultured in complete DMEM medium containing 10% heat-inactivated foetal bovine serum, 1% 100 U/mL penicillin-streptomycin (*v*/*v*), and 1% L-glutamine (*v*/*v*). The cells were maintained in an incubator at 37°C with 5% CO_2_ and subcultured when their growth and proliferation achieved 80% confluency.

### 2.4. The Cell Proliferation Effects of GTN-BG

#### 2.4.1. MTT Assay

The *in vitro* response of the synthesised BG powder on MCF-7 and HMSC (control) was assessed by the Cell Titer 96 Non-Radioactive Cell Proliferation Assay (MTT) (Promega, USA) according to the kit's manual. Briefly, 200 *μ*L of cells at a concentration of 5 × 10^4^ cells/mL was seeded in a 96-well plate and incubated overnight. Then, the cells were treated with different concentrations of BG in a double dilution manner ranging from 0 to 1 mg/mL and incubated for 24, 48, and 72 hours. After incubation times, a volume of 20 *μ*L MTT reagent was added and further incubated for 3 hours in a humidified 5% CO_2_ incubator at 37°C. Then, the media was discarded and 100 *μ*L of stop solution was added to the wells and further incubated for 1 hour. The absorbance was measured at 570 nm with 630 nm as a reference using a microplate reader (BioTek, USA). The effects of GTN-BG on the viability of MCF-7 and HMSC were also assessed by the MTT assay. The cells were treated with 0.8 *μ*g/mL of GTN, which is the IC_50_ values for MCF-7 cells as obtained in our previous study [14] and 0.5 mg/mL of BG, and incubated for 72 hours. Doxorubicin at 0.5 *μ*g/mL was used as a positive control.

#### 2.4.2. PrestoBlue Assay

PrestoBlue® (Thermo Fisher, USA) was used to further examine the proliferation of MCF-7 cells in response to GTN-BG treatment at different doses and incubation times. The procedures were performed according to the manual of the kit. Briefly, the seeded cells at a concentration of 5 × 10^4^ cells/mL were cultured overnight. Following the overnight incubation, the cells were treated with GTN-BG (GTN at IC_25_ (0.4 *μ*g/mL), IC_50_ (0.8 *μ*g/mL), and IC_75_ (1.2 *μ*g/mL) and BG at 0.5 mg/mL). Doxorubicin at 0.5 *μ*g/mL was used as a positive control. The cells were then incubated in a CO_2_ incubator with 5% CO_2_ at 37°C for 24, 48, and 72 hours. A volume of 10 *μ*L of PrestoBlue was added into each well and further incubated for 1 hour. Absorbance at 570 nm and 600 nm was recorded as a reference using a microplate reader (BioTek, USA).

#### 2.4.3. Scratch Wound Assay

The procedure was performed according to the protocol of IncuCyte® Scratch Wound Assay (Sartorius, Germany). The MCF-7 cells at a concentration of 5 × 10^4^ cells/mL were cultured in IncuCyte® ImageLock 96-well microplates and incubated in a CO_2_ incubator with 5% CO_2_ at 37°C until the cells formed a confluent monolayer. Then, the layer was scratched using a WoundMaker™ (Essen BioScience, USA), which is a 96-pin mechanical device designed to create homogeneous 700-800 *μ*m wide wounds in cell monolayers on the plate. The plate was then washed twice with PBS to remove cell debris and subsequently treated with GTN-BG (5 *μ*g/mL GTN+0.5 mg/mL BG). Doxorubicin at 0.5 *μ*g/mL was used as a positive control. The proliferation of cells on the scratch gap was monitored, and the time-lapse images were captured hourly by the IncuCyte ZOOM® system (Essen BioScience, USA) at 10x magnification.

### 2.5. Cell Cycle Analysis

The MCF-7 cells were seeded in 6-well microplates at a concentration of 5 × 10^4^ cells/mL, cultured in medium without serum to synchronise the cell phase, and incubated overnight in a CO_2_ incubator with 5% CO_2_ at 37°C. The treatments with GTN-BG (0.8 *μ*g/mL GTN+0.5 mg/mL BG) and doxorubicin at 0.5 *μ*g/mL (positive control) were performed on the next day in complete growth media, and cells were further incubated for 48 hours. Then, the cells were collected and fixed in 70% ethanol and incubated for 1 hour at 4°C. The staining procedure was performed using the experimental protocol of FxCycle™ PI/RNase staining solution (Thermo Fisher, USA). The fixed cells were spun down at 300 × *g*, and a volume of 500 *μ*L of the staining solution was added to the pellet, followed by incubation at room temperature for 15 to 30 minutes in the dark. The FACSCalibur flow cytometer (BD Biosciences, USA) with CellQuest Pro software was used to analyse cell cycle progression of the stained cells for at least 15,000 events. The data obtained was further analysed using ModFit software.

### 2.6. Annexin V-FITC/PI Staining Assay

The apoptotic events were detected using FITC-Annexin V Apoptosis Detection Kit II (BD Biosciences, USA). The procedures were performed according to the manual of the kit. Briefly, the MCF-7 cells at a concentration of 5 × 10^4^ cells/mL were seeded in 6-well microplates and incubated overnight in a CO_2_ incubator with 5% CO_2_ at 37°C. The cells were treated with GTN-BG (0.8 *μ*g/mL GTN+0.5 mg/mL BG), and doxorubicin at 0.5 *μ*g/mL was used as a positive control. After 48 hours of treatment, both cultures were collected and centrifuged at 300 × *g*. A volume of 100 *μ*L of 1X binding buffer was added to the cell pellet and transferred to a 5 mL round bottom culture tube (BD Biosciences, USA). The cells were then stained with an equal volume of Annexin V-FITC and propidium iodide (PI) (5 *μ*L). The mixture was incubated for 15 minutes in the dark at room temperature. Then, a volume of 400 *μ*L of 1X binding buffer was added to stop the reaction, and the stained cells were analysed by FACSCalibur flow cytometry (BD FACSCalibur flow cytometry, US) using CellQuest Pro software for at least 5,000 events.

### 2.7. Activation of Caspase-3/7, Caspase-8, and Caspase-9

To investigate the activation of caspase-3/7, caspase-8, and caspase-9, the Cell Meter™ Caspase-3/7, Caspase-8, and Caspase-9 Activity Assay Kit (AAT Bioquest®, USA) was used according to the manufacturer's manual. The MCF-7 cells at a concentration of 5 × 10^4^ cells/mL were seeded in 96-well plates. After an overnight incubation, the cells were treated with GTN-BG (0.8 *μ*g/mL GTN+0.5 mg/mL BG) for 48 hours. Doxorubicin at 0.5 *μ*g/mL was used as a positive control. Then, a volume of 100 *μ*L of caspase solution of caspase-3/7 substrate (DEVD-ProRed™), caspase-8 substrate (IETD-R110), and caspase-9 substrate (LEHD-AMC) was individually added and incubated at room temperature for 1 hour in the dark. The fluorescence intensity for caspase-3/7, caspase-8, and caspase-9 was monitored at 535/620 nm, 490/525 nm, and 370/450 nm, respectively, using a fluorometer plate reader (BMG Labtech, Germany).

### 2.8. Statistical Analysis

All experiments were performed in triplicate, and each data represents the mean ± standard deviation. For testing the effects of GTN-BG on MCF-7 cells, the group of samples consists of control (untreated), BG only, GTN only, GTN-BG, and DOX (positive control). Comparisons between the groups were done by using one-way ANOVA, followed by Tukey's posttest for multiple comparisons (^∗^*p* < 0.05, ^∗∗^*p* < 0.01, and ^∗∗∗^*p* < 0.001; ns: not significant) in the MTT cell viability assay. For the cell cycle, the comparison was done using one-way ANOVA, followed by Dunnett's posttest to identify significant differences between the treated and untreated groups (^∗^*p* < 0.05, ^∗∗^*p* < 0.01, and ^∗∗∗^*p* < 0.001), whereas, for the PrestoBlue assay, Annexin V-FITC, and caspase assay, comparisons between the groups were done by using two-way ANOVA, followed by the Bonferroni posttest for multiple comparisons. Significant differences between the treated and untreated groups were marked as ^∗^*p* < 0.05, ^∗∗^*p* < 0.01, and ^∗∗∗^*p* < 0.001, and the significant differences between GTN and GTN-BG treatments were represented as ^∗^*p* < 0.05, ^++^*p* < 0.01, and ^+++^*p* < 0.001.

## 3. Results

### 3.1. Characterisation of Synthesised Sol-Gel-Derived BG 45S5

**Figure 1:**
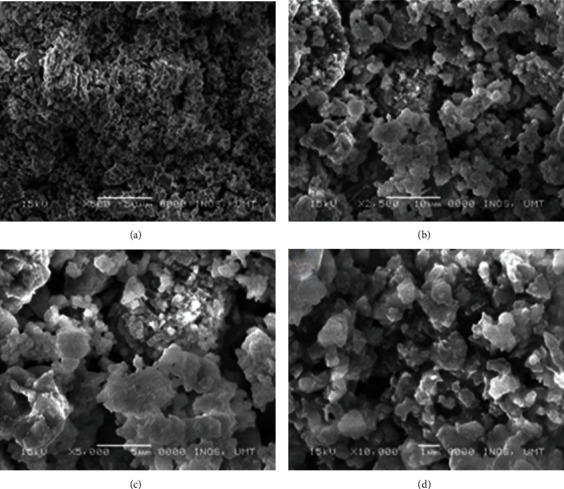
Scanning electron microscopy and SEM micrographs of the bioactive glass 45S from a lower to higher magnification (100 *μ*m to 1 *μ*m; a–d).

**Figure 2:**
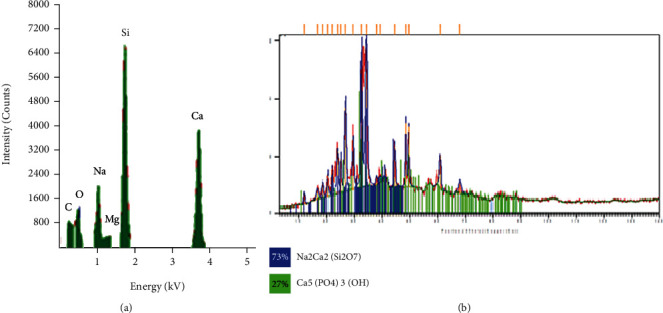
(a) EDS spectrum and (b) XRD pattern of the synthesised BG 45S5 with 73% of pattern similarity to Na_2_Ca_2_(Si_2_O_7_) and 27% to hydroxyapatite.

**Figure 3:**
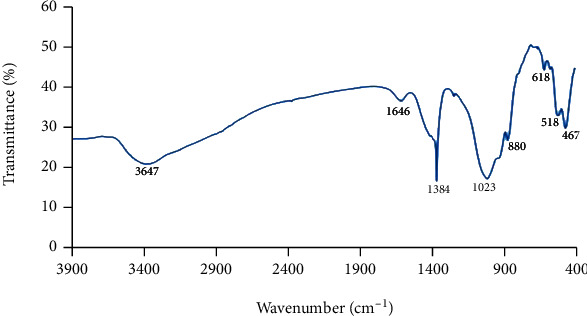
FTIR spectra of the sol-gel-derived BG 45S5.

**Figure 4:**
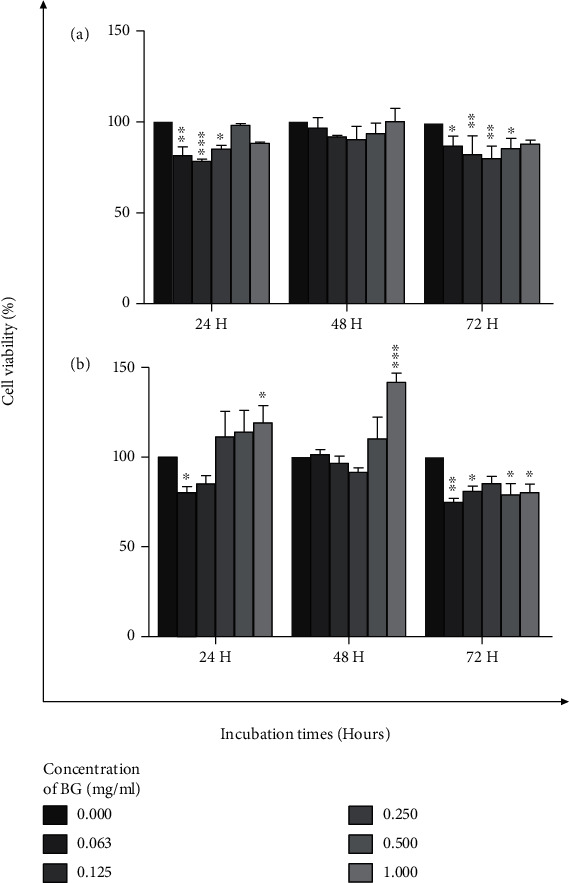
The cytotoxicity of BG against (a) HMSC and (b) MCF-7 cells, at different concentrations from 0 to 1 mg/mL, was assessed by the MTT assay for 24-, 48-, and 72-hour incubation times.

The synthesised BG 45S5 powder has a particle size smaller than 38 *μ*m (mean = 15.24 *μ*m; d10 = 2.22 *μ*m, d50 = 12.1 *μ*m, and d90 = 31.4 *μ*m), which consists mainly of mesopores with a pore size of 126.1255 Å or 12.6 nm, BET surface area of 3.0210 m^2^/g, and pore volume of 0.009526 cm^3^/g. The surface of the glass, as observed in the micrograph, appears rough as shown in Figure 1(a). Micrograph at higher magnification shows the porous texture of the sol-gel-derived material (Figures 1(b)–1(d)). The EDS spectrum presented along with the micrograph confirms the elemental composition of the as-prepared glass. EDS analysis revealed that calcium and silicon elements are the main components of BG nanospheres (Figure 2(a)). Besides, both crystalline and amorphous structures of the material were detected with 73% and 27% similarity to those of Na_2_Ca_2_(Si_2_O_7_) and hydroxyapatite, respectively (Figure 2(b)). As shown in Figure 3, the FTIR spectrum of the sol-gel-derived BG demonstrated the presence of bands at 467 cm^−1^, 518 cm^−1^, 618 cm^−1^, 880 cm^−1^, 1023 cm^−1^, 1384 cm^−1^, 1646 cm^−1^, and 3647 cm^−1^. The different characteristics of the silica network are reflected in the bands: the bands at 467 and 518 cm^−1^ indicate a Si-O-Si band mode; a small band at 880 cm^−1^ is in the range of 940–860 cm^−1^, which indicates Si–O–Si stretching of nonbridging oxygen atoms; the band at 1023 cm^−1^ is in the range of 1100–1000 cm^−1^, indicating Si–O–Si asymmetric stretching of bridging oxygen atoms within the tetrahedra. On the other hand, the band at 1384 cm^−1^ indicates the carbonate group, while the band at 1646 cm^−1^ indicates the occurrence of water absorption at the glass interface. Meanwhile, a broad band located at about 3647 cm^−1^ denotes the hydroxyl group (–OH) or silanol group (Si–OH) vibrations [23, 24]. The synthesised BG was found nontoxic to MCF-7and HMSC cells since no IC_50_ values were detected at concentrations from 0 to 1 mg/mL for all incubation times from 24 to 72 hours (Figure 4). There were some increments in the viability of cells observed in MCF-7 cells at 1 mg/mL for 24 and 48 hours of incubation times. In contrast, 72-hour treatment of BG at 0.063 to 1 mg/mL concentration has significantly decreased the viability of MCF-7 cells. Since BG was found nontoxic to MCF-7 cells as no IC_50_ values were detected at a concentration from 0 to 1 mg/mL, the median BG concentration at 0.5 mg/mL was selected to be combined with GTN.

### 3.2. The Effects of GTN-BG on MCF-7 Cell Proliferation

**Figure 5:**
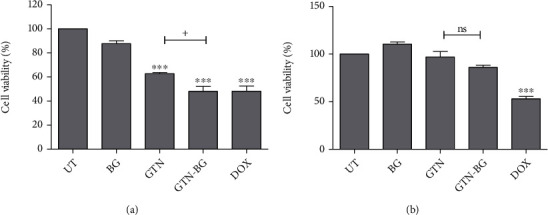
(a) MCF-7 and (b) HMSC cells were treated with GTN at IC_50_ and 0.5 mg/mL of BG. Cell viability was assessed *via* the MTT assay. All experiments were done in triplicate, and the data is represented by means and standard deviations. Comparisons between the groups of different treatments were done by using 1-way ANOVA, followed by Tukey's posttest for multiple comparisons. Not significant is denoted as ns; the significant difference in the treated cells as compared to untreated cells is marked as ^∗^*p* < 0.05, ^∗∗^*p* < 0.01, and ^∗∗∗^*p* < 0.001, whereas the significant difference between GTN and GTN-BG treatments is represented as ^+^*p* < 0.05, ^++^*p* < 0.01, and ^+++^*p* < 0.001.

The proliferation of GTN-treated MCF-7 cells at IC_50_ values (0.8 *μ*g/mL) with and without the addition of BG (0.5 mg/mL) was investigated. In comparison to the untreated cells, a single GTN treatment reduced nearly half of the cell population as expected (Figure 5(a)). However, there was a significant difference (*p* < 0.001) between the treatment of GTN-BG and GTN, indicating that the combination was more effective in killing MCF-7 cells. The combined effect of GTN-BG on cell proliferation was also tested in HMSC using the same dose used for MCF-7 cells. As shown in Figure 5(b), there was no significant difference observed between the cells treated with BG, GTN, or GTN-BG and the untreated HMSC cells.

**Figure 6:**
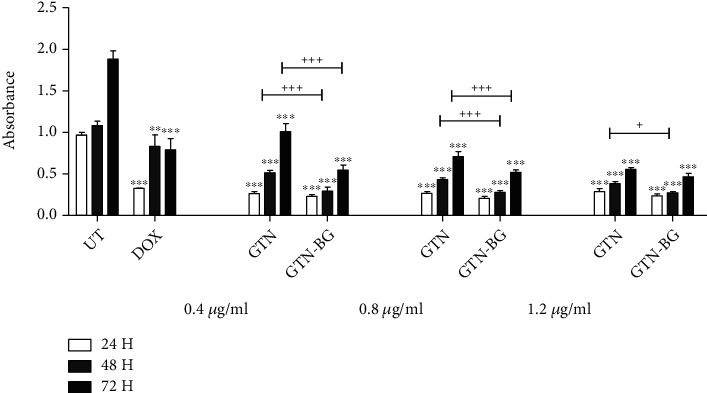
The inhibitory effects of GTN and GTN-BG at IC_25_, IC_50_, and IC_75_ in MCF-7 following 24, 48, and 72 hours of incubation time. All experiments were done in triplicate, and the data is represented by means and standard deviations. The comparisons between the groups were done by using two-way ANOVA, followed by the Bonferroni posttest for multiple comparisons. Significant differences between the treated (DOX, GTN, and GTN-BG) and untreated (UT) groups were marked as ^∗^*p* < 0.05, ^∗∗^*p* < 0.01, and ^∗∗∗^*p* < 0.001, and the significant differences between GTN and GTN-BG treatments were represented as ^∗^*p* < 0.05, ^++^*p* < 0.01, and ^+++^*p* < 0.001.

The effects of the combination of GTN-BG on the proliferation of MCF-7 cells in different dosages (IC_25_, IC_50_, and IC_75_) and incubation times (24, 48, and 72 hours) were further investigated. As shown in Figure 6, the inhibitory effects of GTN-BG in MCF-7 cells were observed in a dose- and time-dependent manner, which demonstrates that higher concentrations and longer incubation time have significantly decreased the cell viability. Besides, the GTN-BG treatment was found more effective compared to the single GTN treatment. Figure 6 emphasises the inhibitory effects of each treatment. In comparison to the untreated cells, the proliferation of cells treated with GTN, GTN-BG, and DOX was significantly inhibited for all incubation times and at all concentrations of GTN and GTN-BG tested. Treatment with GTN-BG significantly decreased cell proliferation, more efficiently than GTN treatment at both concentrations of IC_25_ and IC_50_ and incubation time of 48 and 72 hours. At a concentration of IC_75_, treatment with GTN-BG was more effective than GTN alone only for 48 hours.

**Figure 7:**
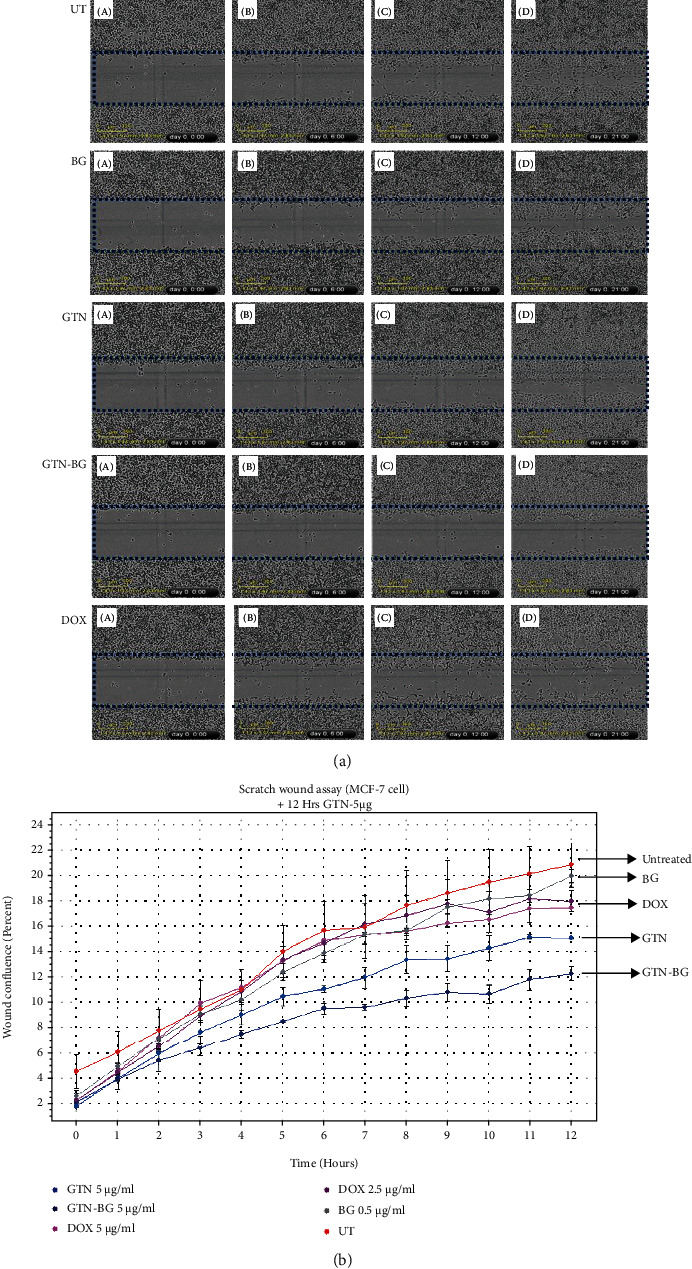
(a) Confluent monolayer of MCF-7 cells was mechanically wounded (scratch assay), leaving two wound edges separated by a 700-800 *μ*m wide void (gap). The proliferation of untreated MCF-7 cells and the cells treated with BG, GTN, GTN-BG, and DOX on the void was monitored using the IncuCyte ZOOM® system at 10x magnification. The time-lapse images were taken at 0, 6, 12, and 21 hours, which are represented as A, B, C, and D, respectively. (b) The real-time proliferation of MCF-7 cells on the gap and the percentage of wound confluence were measured by using the IncuCyte™ ZOOM Live Cell System (Essen BioScience, USA) for up to 12 hours.

Figure 7(a) illustrates the time-lapse images of untreated MCF-7 cells and MCF-7 cells treated with BG, GTN, GTN-BG, and DOX for 0, 6, 12, and 21 hours. Proliferation of MCF-7 cells on the gap was monitored using the IncuCyte ZOOM® system at 10x magnification. The confluent monolayer of untreated and BG-treated MCF-7 cells took less than 21 hours to close the gap, whereas the addition of GTN, GTN-BG, and DOX to the confluent monolayer of cells affects the cell proliferation by delaying the progression of gap closure by the cells. In comparison with GTN, the percentage of confluency in GTN-BG treatment was decreased with the prolonged progression of MCF-7 cells in the gap (Figure 7(b)).

### 3.3. Analysis of Cell Cycle Progression

**Figure 8:**
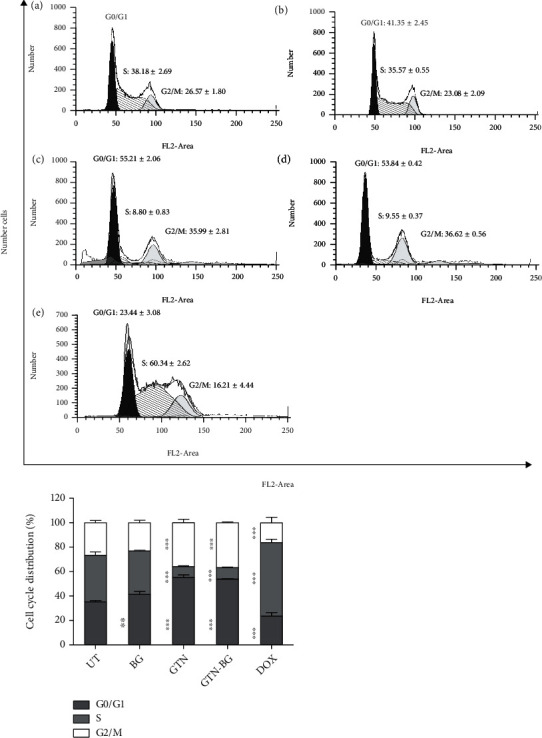
DNA histogram of MCF-7 cells in (a) untreated, (b) BG-treated, (c) GTN-treated, (d) GTN-BG-treated, and (e) DOX-treated groups after 48 hours of exposure time. The bar graph shows the quantitative data based on DNA histograms. Comparisons between the different treatment groups were done by using 1-way ANOVA, followed by Dunnett's posttest to detect any significant differences between the treated and untreated cells (^∗^*p* < 0.05, ^∗∗^*p* < 0.01, and ^∗∗∗^*p* < 0.001).

Figure 8(a) illustrates the DNA histograms of the MCF-7 cell cycle after 48 hours of incubation time with the respective quantitative data presented in the bar graph (Figure 8(b)). The GTN and GTN-BG treatments have caused accumulation in the G0/G1 and G2/M phase, followed by a reduction in the percentage of cells in the S phase. In contrast, the DOX-treated cells exhibited accumulation in the S phase, which significantly decreased in the G2/M and G0/G1 phases. The percentage of cells undergoing G0/G1 in untreated cells was 35.24 ± 1.14, which increased to 55.21 ± 2.06 and 53.84 ± 0.42 in the cells treated with GTN and GTN-BG, respectively, but the percentage dropped to 23.44 ± 3.08 in DOX-treated cells. The percentage of untreated cells in the S phase was 38.18 ± 2.69, which decreased to 8.80 ± 0.83 and 9.55 ± 0.37 in the cells treated with GTN and GTN-BG, respectively, but the percentage increased to 60.34 ± 2.62 in DOX-treated cells. Approximately, the percentage of cells undergoing the G2/M phase was 26.57 ± 1.80 in untreated cells, which increased to 35.99 ± 2.81 and 36.61 ± 0.56 in GTN- and GTN-BG-treated cells, respectively. On the other hand, a significant reduction in cells of the G2/M phase was observed in DOX-treated cells with a calculated percentage of 16.21 ± 4.44.

### 3.4. Induction of Apoptosis

**Figure 9:**
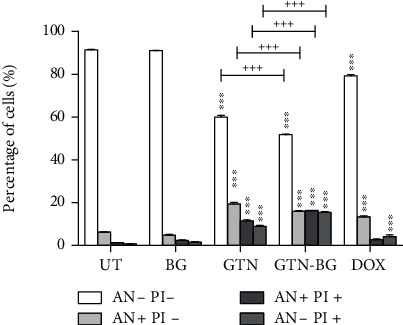
The percentage of stained MCF-7 cells following Annexin V-FITC/PI analysis by using flow cytometry for 48 hours. All experiments were performed in triplicate, and the data is represented by means ± standard deviations. Comparisons between the different treatment groups were done by using 2-way ANOVA, followed by the Bonferroni posttest for multiple comparisons. The significant difference between the treated and untreated cells is marked as ^∗^*p* < 0.05, ^∗∗^*p* < 0.01, and ^∗∗∗^*p* < 0.001, whereas the significant difference between GTN- and GTN-BG-treated cells is represented by ^+^*p* < 0.05, ^++^*p* < 0.01, and ^+++^*p* < 0.001.

The mode of cell death in MCF-7 cells in response to different treatments was determined by the Annexin V-FITC assay. The percentage of mode of cell death in stained cells was calculated based on four different events: viable cells (An-/PI-), early apoptotic cells (An+/PI-), late apoptotic cells (An+/PI+), and necrotic cells (An-/PI+). As shown in Figure 9, the treatments with GTN and GTN-BG have significantly decreased the percentages of viable cells of MCF-7 cells. Conversely, there was a significant (*p* < 0.001) increment in the percentages of early and late apoptotic cells in both treatments. In comparison with GTN, treatment with GTN-BG significantly decreased the percentages of viable and early apoptotic cells but increased the percentage of late apoptotic cells.

### 3.5. Activation of Caspase-8, Caspase-9, and Caspase-3/7

**Figure 10:**
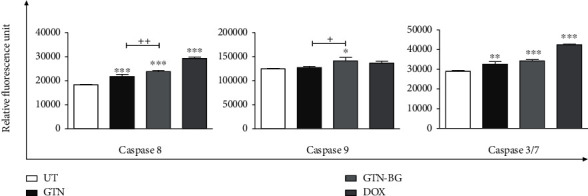
Activation of caspase-8, caspase-9, and caspase-3/7 in MCF-7 cells after 48 hours. All experiments were performed in triplicate, and the data is represented by means ± standard deviations. Comparisons between the groups of different treatments were done by using 1-way ANOVA, followed by the Bonferroni posttest for multiple comparisons. The significant difference between the treated and untreated cells is marked as ^∗^*p* < 0.05, ^∗∗^*p* < 0.01, and ^∗∗∗^*p* < 0.001, whereas the significant difference between GTN- and GTN-BG-treated cells is represented as ^+^*p* < 0.05, ^++^*p* < 0.01, and ^+++^*p* < 0.001.

The activation of initiator caspases for both extrinsic and intrinsic pathways, namely, caspase-8, caspase-9, and caspase-3/7, was studied in MCF-7 cells in response to GTN, GTN-BG, and DOX treatments. As shown in Figure 10, all treatments have significantly activated caspase-8 and caspase-3/7 compared to the untreated cells. Treatment with GTN-BG significantly activated all caspases in the cells compared to the single treatment with GTN. The activation of caspase-9 was only significantly observed in cells treated with GTN-BG. Among all caspases, caspase-8 was activated the most, followed by caspase-3/7 and caspase-9.

## 4. Discussion

GTN has been reviewed as a potent cytotoxic agent for the induction of apoptosis in many cancer cell lines [13]. The promising anticancer properties of GTN have been studied in several breast cancer cells, including SK-BR-3, MDA-MB-231, and MCF-7 [21, 25–28]. In SK-BR-3 cells, the GTN-induced apoptosis was associated with autophagy *via* p-p38 and p-JNK1/2 upregulation and Akt downregulation [25], whereas nonapoptotic cell death mechanisms, namely, necroptosis and anoikis, induced by GTN were reported in human invasive breast cancer cells MDA-MB-231 [26]. Not only limited to its pure compound, research on the effects of GTN's derivatives as well as its potential combination with other biomaterials was also reported. A study conducted by Boonmuen et al. found that 5-acetyl goniothalamin (5GTN), a natural derivative of GTN, was more potent than GTN in mediating the toxicity towards MCF-7 and MDA-MB-231 breast cancer cells [27]. Besides, a fluorescent 2,1,3-benzothiadiazole-containing goniothalamin derivative, BTD−GTN [1] hybrid, was successfully synthesised in an investigation to gain insights into the subcellular localisation and mechanism of action of MDA-MB-231 cells, which might involve a cascade of events, starting with their interaction with mitochondria [29]. In a recent study, a polymeric nanosystem, in which the racemic mixture of GTN (rac-GTN) was encapsulated in pH-responsive acetalated dextran (Ac-Dex) nanoparticles (NPs), has been developed to improve the pharmacokinetic behaviour and selectivity of GTN against cancer cells including MCF-7 and MDA-MB-231 cells [30].

Multiple polymeric nanoparticles, liposomes, and micelles have demonstrated great potential in drug delivery. However, the potentials of these materials are challenged by their poor bioavailability and biodegradability, instability in the circulation, and inadequate distribution in the tissue [31]. On the other hand, sol-gel bioactive glass is known to be bioactive, biocompatible, and degradable; therefore, there is no requirement for a second surgical procedure to remove the material from the body. Bioactive glass (BG) with controlled diameter serves as an ideal carrier for the delivery of anticancer in the body. One of the approaches proposed is by loading the anticancer drugs directly into the mesoporous bioactive glass (MBG) to produce local chemotherapeutic effects. The potential development of BG as a drug carrier has been reported for several anticancer drugs including doxorubicin, imatinib, and 5-fluorouracil [11, 12, 32, 33]. The third drug has been extensively used clinically to treat various types of cancer. Despite its efficacy in killing cancer cells, the only drawback is that it is easily metabolised due to the short biological lifespan. A study done by El Kadi et al. revealed the potential use of bioactive glass nanoparticles (SiO_2_-CaO-P2O_5_) as a delivery system for 5-fluorouracil [33]. Their findings demonstrate that the BG was able to sustain the release of 5-fluorouracil for more than 32 days, which could prevent cancer recurrence after resection. Another approach is through the modification of the BG surface by incorporating anticancer metals to improve its specificity towards the receptors of cancer cells. The examples include terbium (Tb), holmium (Ho), 153Sm-ethylenediaminetetrame thylphosphonic acid (153Sm-EDTMP), and yttrium. Interestingly, a recent research studied the combined effects of copper- (Cu-) doped BGs and the surface-modified BG, which exhibited both photothermal and chemotherapeutic activities towards bone tumours [8]. Apart from these approaches, nanoscale HA-based biomaterials have been found capable of inhibiting proliferation and inducing apoptosis in various cancer cells including breast, colon, gastric, osteosarcoma, and liver cancer cells [34–40].

The present study was designed to examine the combined effects of GTN and BG (GTN-BG) in MCF-7 cells in comparison with a single GTN treatment to postulate whether the combination could enhance the antitumour effects. The BG was successfully prepared using the sol-gel method, and the characterisation was performed to understand its properties and predict its biological performance in this study. The synthesised BG 45S5 is classified as a fine powder with high surface area that enables high dissolution rate. Moreover, it was reported that the use of fine powders has enhanced the deposition level of the Ca-P layer on the glass surface and material degradation and resorption rates [41]. Besides, the synthesised BG 45S5 comprises the rough surface and mainly consists of mesopores that formed as a result of gel formation. Due to the mesoporous texture and high surface area, which can adsorb a range of substances including proteins and cells, the sol-gel method has become an option for several biomedical applications [42].

The cellular response was first evaluated by looking at the inhibitory effects of GTN-BG in MCF-7 cells. In this study, HMSC were used as the control due to its multipotent differentiation capacity into at least three mesodermal lineages including chondrocytes, osteoblasts, and adipocytes [43]. To date, reports have shown an increasing number of MSC-based clinical trials for diseases associated with wound healing, inflammation, and degeneration in various organs and tissues [44]. HMSC also have the potential to differentiate into nonmesodermal lineages, specifically neurons and glia [45]. Moreover, the role of HMSC as a gene carrier in various types of cancer cells for the application of cancer therapy indicates its versatility with distinct cellular properties [46]. We have demonstrated that the combination of GTN-BG was more potent than GTN in inhibiting the proliferation of MCF-7 cells, while the control cells of HMSC were not affected. The analysis of cell cycle progression indicated that the cell cycle arrest occurred at similar phases in both GTN- and GTN-BG-treated cells, which was at G0/G1 and G2/M phases. Concurrently, there was a reduction in the percentage of cells at the S phase. Other studies have demonstrated that GTN arrested the cell cycle at G0/G1 in MCF-7 [28], G2/M in MDA-MB-231 [21], and S phase in Hela cells [47]. The relative contribution of G1 and G2/M arrests may vary according to the cell line, treatment time, and dosage. The G1 arrest was reported to be associated with the reduced expression of cyclin D1 and CDK4 mRNA, in addition to the downregulation of CDK2. Meanwhile, the G2/M phase arrest was believed to be related to the downregulation of CCNB1, CCNB2, and CDK1 [27].

The mode of cell death was determined by the Annexin V-FITC assay. We have demonstrated that both GTN and GTN-BG treatments induced apoptosis in MCF-7, as the percentages of both early and late apoptotic cells were significantly increased after the treatments. In comparison with GTN, the cells treated with GTN-BG cells produced a lower number of viable cells but a higher number of late apoptotic cells than those of GTN. Although the percentage of necrotic cells increased in both treatments, apoptosis was considered the primary mode of cell death as the total percentage of apoptotic cells was higher than that of the necrotic cells.

Activation of caspases is the key event in apoptosis, since caspases initiate irreversible processes of cell death [48]. Cells undergo apoptosis through two major pathways, the extrinsic (death receptor) and intrinsic (mitochondrial) pathways; both end with the execution phase, which is regarded as the final pathway of apoptosis [48, 49]. Activation of caspases, namely, caspase-8, caspase-9, and caspase-3/7, was examined in this study. Caspase-8 is involved in the extrinsic pathway of apoptosis, while the other two caspases are involved in the intrinsic pathway of apoptosis. In this study, we have found that GTN-BG significantly activated caspase-8 and caspase-9 that are responsible for the extrinsic and intrinsic pathways of apoptosis, respectively. The caspase cascade was further triggered through the activation of caspase-3/7. Theoretically, the activation of caspase-8 is mediated by cell surface death receptors, such as Fas, tumour necrosis factor receptor, and TRAIL receptors. Cell death ligand triggers oligomerisation of the receptors and recruitment of the adaptor proteins, Fas-associated death domain (FADD) and caspase-8, to form death-inducing signalling complex (DISC), whereas the activation of caspase-9 is initiated by the release of cytosolic cytochrome from the mitochondria to the cytoplasm and its binding to the apoptosis protease-activating factor 1 (Apaf-1) and procaspase-9; the binding generates an intracellular DISC-like complex known as “apoptosome,” which later activates caspase-9 [49]. The culmination of both extrinsic and intrinsic pathways ends at the point of the execution phase, which is considered the final pathway of apoptosis. Caspase-3, caspase-6, and caspase-7 function as an effector or “executioner” caspases, cleaving various substrates that drive the terminal events of the programmed cell death [48, 50].

The present study demonstrated the relatively high inhibitory effects of GTN-BG towards the proliferation of MCF-7 cells and activation of caspase-8, caspase-9, and caspase-3/7 in comparison to GTN. It is important to note that both treatments of GTN-BG and GTN have activated the same cellular death program known as apoptosis, which resulted in the cell cycle arrest at G0/G1 and G2/M phases. Unfortunately, the understanding of the mechanism of BG's action in enhancing the antitumour effect of GTN in MCF-7 remains unclear. However, we strongly believe that such enhancement might be due to the physicochemical properties of BG. The porous structure with a high surface area of the sol-gel-derived BG makes entrapment of molecules or drugs inside the pores more effective than other materials, which enhances the delivery of the drug to the cells [51]. Upon contact with biological fluids, dissolution of BG triggers the release of not only drugs but also ions. Consequently, BG dissolves gradually and the released ions would stimulate the growth of the hydroxyapatite (HA) layer on its surface [52–56]. Some studies have shown that nanoscale HA-based biomaterials can inhibit proliferation and induce apoptosis in several types of cancer cells including breast cancer cells [34–40]. The production of intracellular reactive oxygen species and activation of p53 that are responsible for DNA damage and apoptosis were reported in MCF-7 cells cocultured with HA nanoparticles [40].

The release of ionic dissolution products from the BG such as Ca^2+^, Na^+^, PO_4_^3−^, and Si^4+^ has been shown to result in the rise of pH and osmotic pressure in its vicinity, which created an efficient antibacterial effect [57, 58]. The role of extracellular pH in treating cancer cells has been emphasised in many studies. It is known that the extracellular pH of cancer cells is more acidic compared to that of the normal cells, due to the excess of anaerobic glycolysis. Thus, increasing the extracellular pH may be a good strategy to inhibit the progression of tumour cells [59–61].

## 5. Conclusion

Inhibition of cell proliferation, cell cycle arrest at G0/G1 and G2/M phases, and induction of apoptosis in MCF-7 cells in the treatment of GTN-BG may be due to the toxicity of goniothalamin against the cells and the microenvironment provided by the unique properties of BG. It is important to highlight that the GTN-BG treatment was more potent than the single treatment with GTN. However, the exact mechanism remains unclear, and there are still concerns regarding the efficacy of these biomaterials to be used as an effective cancer therapeutic drug, which should be investigated *in vitro* and *in vivo*. Most importantly, the potential adverse effects associated with the use of the proposed biomaterial should be extensively studied to ensure its safety prior to human use.

## Data Availability

Data sharing is not applicable to this article as no datasets were generated or analysed in the current study.

## Abbreviations

Ac-Dex: Acetalated dextran
BG: Bioactive glass
DISC: Death-inducing signalling complex
DOX: Doxorubicin
FADD: Fas-associated death domain
FTIR: Fourier transform infrared spectroscopy
GTN: Goniothalamin
HA: Hydroxyapatite
HMSC: Human bone marrow-derived mesenchymal stem cells
MSC: Mesenchymal stem cells
MBG: Mesoporous bioactive glass
NP: Nanoparticles
SEM: Scanning electron microscope
XRD: X-ray diffraction analysis.
